# Chitin nanowhiskers/octacalcium phosphate synergistically reinforced chitosan hydrogel for enhanced bone regeneration

**DOI:** 10.1186/s13036-026-00647-8

**Published:** 2026-03-18

**Authors:** Mingdong Yan, Yanjing Ou, Ruimin Liu, Changfu Xie, Jiang Chen

**Affiliations:** 1 Fujian Key Laboratory of Oral Diseases and Fujian Provincial Engineering Research Center of Oral Biomaterial and Stomatological Key Laboratory of Fujian College and University, School and Hospital of Stomatology, Fujian Medical University, Fuzhou, 350002 China; 2 Department of Oral Implantology, Affiliated Stomatological Hospital of Fujian Medical University, Fuzhou, 350002 China; 3 ORAL Center, Fujian Provincial Governmental Hospital (Affiliated Hospital of Fujian Health College), Fuzhou, 350003 China

**Keywords:** Chitosan, Chitin nanowhiskers, Octacalcium phosphate, Bone regeneration

## Abstract

The repair of jawbone defects faces critical challenges, such as the mechanical-bioactivity imbalance and limited bioactivity of traditional scaffold materials. This study proposes a “mechanical reinforcement–bio-mineralization” synergistic strategy to construct a chitin nanowhiskers (CHWs)/octacalcium phosphate (OCP) co-modified chitosan (CS) composite hydrogel (CS/CHWs-OCP) and systematically investigates its bone regeneration efficacy. Results demonstrate the successful fabrication of CS/CHWs-OCP hydrogels with gradient OCP loading (3%–12% wt). The hydrogels exhibit three-dimensional interconnected porous structures, favorable swelling properties, and effective protein loading/sustained-release capabilities. In vitro experiments reveal that CS/CHWs-OCPs significantly promote osteogenic differentiation of bone marrow mesenchymal stem cells (BMSCs) and angiogenic differentiation of EA.hy926 cells, with the 12 wt% OCP group showing the highest alkaline phosphatase (ALP) activity. Micro-CT 3D reconstruction and histological analysis demonstrate that the 12 wt% OCP group exhibits optimal calvarial defect repair capacity, with its new bone volume fraction (BV/TV: 57.2 ± 10.2%) at 8 weeks being significantly superior to both the blank control and OCP-free hydrogel groups. In conclusion, the developed CS/CHWs-OCP hydrogel provides an effective solution for bone defect regeneration.

## Introduction

The repair of maxillofacial bone defects is a critical research direction in regenerative medicine. Clinically common etiologies such as periodontal disease, traumatic bone defects, congenital maxillofacial deformities, and post‑tumor resection bone loss often lead to insufficient bone volume at implant restoration sites, significantly compromising oral functional rehabilitation. An ideal bone regeneration scaffold must possess a three-dimensional porous structure to provide cell colonization space while meeting the dual requirements of mechanical support and bioactivity. However, existing scaffold materials generally suffer from technical limitations, including inadequate mechanical strength and insufficient osteogenic/angiogenic induction capacity, which hinder their clinical translation.

Chitosan (CS), owing to its excellent biocompatibility, in vivo degradability, inherent antibacterial properties, and osteoinductive potential, has been widely used in constructing bone repair hydrogels [1, 2]. However, pure CS hydrogels exhibit drawbacks such as low elastic modulus and poor deformation resistance, making them unsuitable for the mechanical demands of load‑bearing bone regeneration. Various fillers, including diatom biosilica [3] and cellulose nanofibers [4], have been employed to enhance the mechanical properties of CS gels.

Chitin nanowhiskers (CHWs) exhibit a needle‑like or rod‑like morphology with a high aspect ratio (greater than 10) and a large specific surface area, boasting a longitudinal modulus of up to 150 GPa. Studies have shown that CHWs, as nanoscale reinforcing fillers, can effectively enhance the compressive modulus and fatigue resistance of polymer matrices [5–10], providing new insights for developing high‑strength hydrogels.

CHWs demonstrate distinct advantages as fillers for CS hydrogels, particularly in biocompatibility, mechanical reinforcement, and structural synergy. Therefore, this study introduces CHWs as a mechanical reinforcing phase.

Octacalcium phosphate (OCP), a biologically derived calcium phosphate compound, plays a dual role in bone metabolism: (1) as a precursor of hydroxyapatite (HA), it participates in bone matrix mineralization through phase transformation [11–13]; and (2) it promotes the adhesion, proliferation, and osteogenic differentiation of bone marrow mesenchymal stem cells (BMSCs) [14]. Notably, OCP degradation kinetics exhibit spatiotemporal coupling with osteogenesis—osteoclasts create a localized acidic microenvironment by secreting acids and enzymes, which may promote the dissolution of OCP, while the released calcium and phosphate ions further stimulate the expression of angiogenic factors (e.g., VEGF), enhancing vascularization in defect areas [13, 15, 16]. However, OCP’s inherent crystal brittleness and lack of bioactive molecule‑controlled release limit its standalone application, necessitating the development of composite scaffold systems that integrate mechanical stability with sustained bioactive component release.

**Scheme 1:**
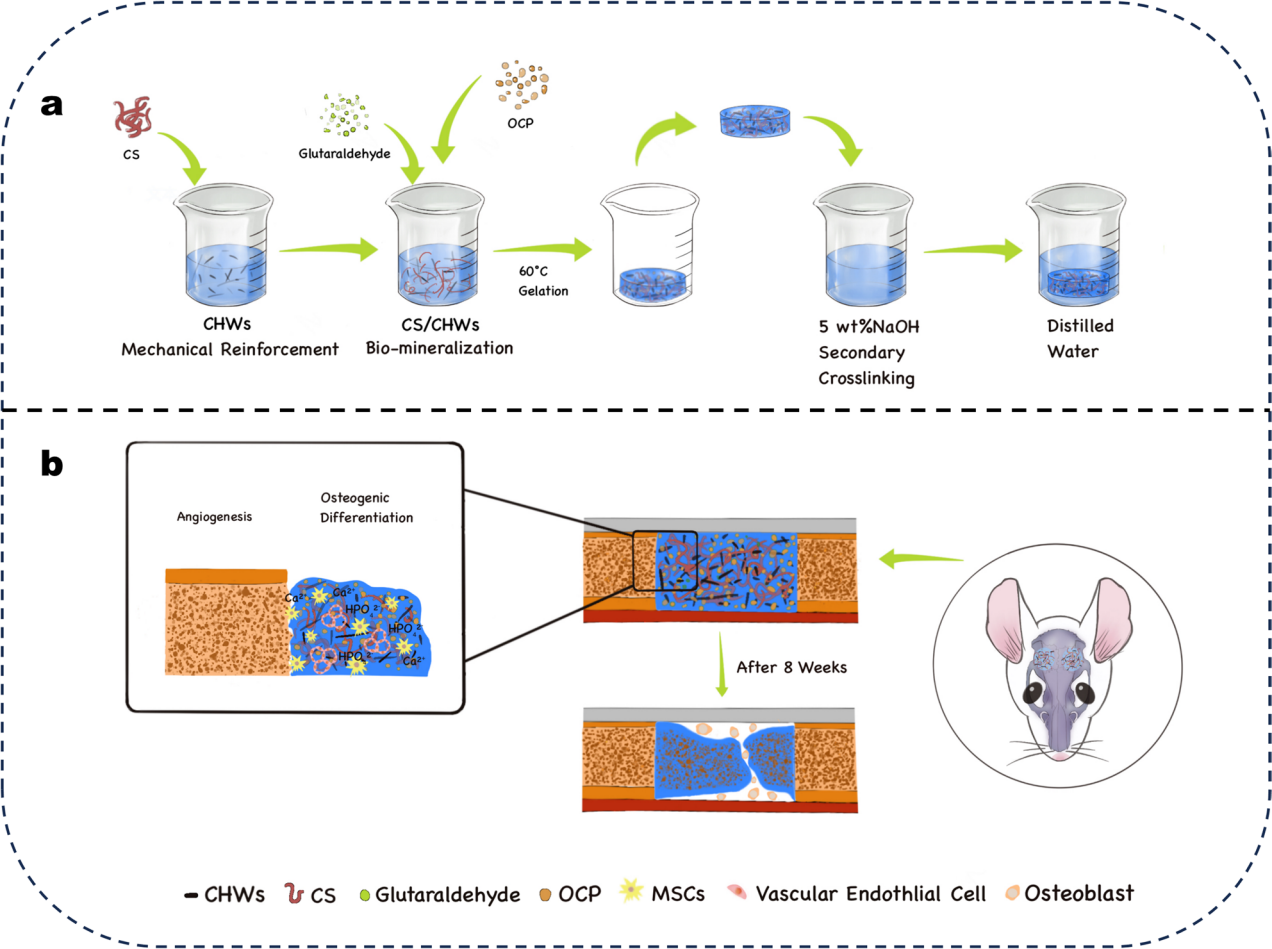
Schematic illustration of (**a**) the fabrication process of CS/CHWs‑OCP composite hydrogels and (**b**) their osteogenic potential in vivo

This study addresses the challenge of imbalance between mechanical properties and bioactivity in materials for bone defect repair, innovatively proposing a synergistic strategy of “mechanical reinforcement–biomineralization.” A chitosan (CS) hydrogel co‑modified with chitin nanowhiskers (CHWs) and octacalcium phosphate (OCP) was constructed (CS/CHWs‑OCP). Although previous studies [3, 4] have used regenerated cellulose nanofibers or diatom biosilica to enhance chitosan‑based materials, most have focused on improving a single property or lacked validation spanning from in vitro to in vivo experiments. This study innovatively proposes a “mechanical reinforcement–bio‑mineralization” synergistic strategy: (1) constructing CHWs‑reinforced CS‑based hydrogels (CS/CHWs) to enhance mechanical properties via physical entanglement between nanofibers and polymer chains; and (2) incorporating highly bioactive OCP particles to develop a ternary CS/CHWs/OCP composite hydrogel, leveraging OCP’s ion‑release capability to confer dynamic mineralization capacity (Scheme 1). The study systematically investigates the effects of varying OCP loading (3–12 wt%) on material morphology, swelling behavior, and protein release. Furthermore, the osteogenic efficacy of the composite scaffold is evaluated through: (1) BMSC osteogenic differentiation (ALP activity assay, Alizarin Red staining); (2) EA.hy926 angiogenic potential; and (3) rat critical‑sized calvarial defect models. This research provides a theoretical foundation for developing novel bone repair materials.

## Materials and methods

### Materials

Chitin powder and chitosan were purchased from Golden‑Shell Pharmaceutical Co., Ltd. (Zhejiang, China). Octacalcium phosphate (OCP) was purchased from Shanghai Aladdin Biochemical Technology Co., Ltd. (China). Glutaraldehyde and other chemical reagents were purchased from Sinopharm Chemical Reagent Co., Ltd. (China).

### Preparation of chitin nanowhiskers (CHWs)

Chitin powder was dispersed in 5 wt% NaOH solution and stirred at 25 °C for 12 h to remove residual proteins, followed by washing with distilled water until neutral pH. The treated chitin was then dispersed in 7 vol% HCl aqueous solution and stirred at 25 °C for 12 h to eliminate mineral salts, followed by neutralization with distilled water. The purified chitin was dried and stored in a desiccator before use.

The purified chitin was partially deacetylated by stirring in 33 wt% NaOH aqueous solution at 90 °C for 3 h. The resulting chitin was washed with distilled water to neutrality and freeze‑dried to obtain partially deacetylated chitin powder. The partially deacetylated chitin was dispersed in 0.1 mol/L acetic acid aqueous solution at a weight ratio of 1:500 (chitin: acetic acid solution) and mechanically stirred for 24 h to protonate the partially deacetylated ‑NH₂ groups. Subsequently, the protonated chitin‑acetic acid solution was ultrasonically treated using a probe sonicator under ice‑bath conditions. The resulting suspension was centrifuged at 8000 rpm for 10 min at 4 °C, and the supernatant was collected as the CHWs suspension. The concentration of the CHWs suspension was determined by freeze‑drying.

### Preparation of CS/CHWs‑OCP Composite Hydrogels

#### Control group hydrogel (CS/CHWs)

A total of 0.3 g chitosan was dissolved in 10 g of 0.5% CHWs suspension. After complete dissolution, 0.01 g glutaraldehyde (crosslinker) was added without OCP (denoted as G). The solution was transferred to a Petri dish, covered with plastic wrap, and reacted in a 60 °C oven for 3 h. The formed hydrogel was then immersed in 5 wt% NaOH aqueous solution for secondary crosslinking and washed with distilled water to neutrality.

#### Experimental group hydrogels (CS/CHWs‑OCP)

A total of 0.3 g chitosan was dissolved in 10 g CHWs suspension. After complete dissolution, 0.01 g glutaraldehyde was added, followed by different amounts of OCP. The mixture was thoroughly stirred and centrifuged to remove air bubbles. The solution was transferred to a Petri dish, covered with plastic wrap, and reacted in a 60 °C oven for 3 h. The formed hydrogels were then immersed in 5 wt% NaOH aqueous solution for secondary crosslinking and washed with distilled water to neutrality.

Based on the mass percentage of OCP added, the composite hydrogels were named as follows: composite hydrogel containing 3 wt% OCP (hereinafter referred to as CG3), composite hydrogel containing 6 wt% OCP (CG6), composite hydrogel containing 9 wt% OCP (CG9), and composite hydrogel containing 12 wt% OCP (CG12).

### Material characterization

The chemical characteristics of each hydrogel group were analyzed using Fourier‑transform infrared spectroscopy (FTIR; Nicolet iS5, Thermo Scientific, USA) and X‑ray diffraction (XRD; D8 Advance, Bruker AXS GmbH, Germany). The morphological features of the materials were examined by scanning electron microscopy (SEM; TESCAN, Czech Republic).

### Mechanical testing

The samples were precisely cut into cylindrical shapes with a diameter of 1.5 cm and a height of 1.5 cm. Uniaxial compression tests were performed using an LR5KPlus mechanical testing machine (Lloyd Instruments, UK) under controlled temperature conditions at 25 °C.

### Swelling behavior of hydrogels

The mass change before and after swelling, as well as the time required to reach equilibrium, were measured using the wet weight method. The swelling experiment was conducted as follows:

Freeze‑dried hydrogels (5 mm diameter, *n* = 3) were immersed in 1 mL of PBS (0.01 mol/L, pH 7.4) and retrieved at 10 s, 20 s, 30 s, 40 s, 50 s, 1 min, 2 min, 1 h, and 2 h. Surface liquid was removed with filter paper before weighing to assess mass increase. The swelling ratio was calculated as: Swelling ratio = Wₛ / W₀ (where Wₛ represents the mass of swollen hydrogel, and W₀ denotes the initial mass of the hydrogel).

### Protein release assay

Lyophilized hydrogels (equal mass) were placed in a 96‑well plate, loaded with 200 µL of BSA solution (5 mg/mL), and incubated at room temperature for 24 h. After removing the BSA solution, 100 µL PBS was added. Supernatants were collected at 1, 2, 4, 8, 16, 24, and 32 days, with immediate replenishment of 100 µL fresh PBS. Protein concentration in the collected PBS was quantified using the BCA assay.

### Isolation and culture of BMSCs

BMSCs were isolated from the femurs of Sprague‑Dawley (SD) rats via whole bone marrow adherence and expanded in vitro for subsequent experiments. Cells from passages 3–5 were used in all studies.

### Cell proliferation assay

Sterilized hydrogels were placed in 24‑well plates, and BMSCs were seeded at 15,000 cells/well. Cultures were maintained in a 5% CO₂ incubator. Cell proliferation activity was assessed at 1, 3, and 5 days using CCK‑8 kits.

### In vitro osteogenic and angiogenic properties of hydrogels

#### Alkaline phosphatase (ALP) activity and alizarin red staining

BMSCs were cultured in osteogenic induction medium (Cyagen Biosciences, Guangzhou, China) to induce osteogenic differentiation.

ALP activity was quantitatively measured after 7 days of osteogenic induction using a commercial kit (Beyotime Biotechnology, China), while Alizarin Red staining was performed at day 14 to evaluate the osteogenic differentiation capacity of BMSCs.

##### ALP activity

Definition of alkaline phosphatase activity unit: One unit of ALP activity (DEA unit) was defined as the amount of enzyme required to hydrolyze 1 µmol of p‑nitrophenylphosphate substrate per minute in diethanolamine (DEA) buffer (pH 9.8) at 37 °C to produce p‑nitrophenol.

The ALP activity in samples was calculated as follows:

$${\rm{ALP\,activity}}\left( {{\rm{nmol/\mu g/min}}} \right){\rm{ = A / }}\left( {{\rm{V \times T \times B}}} \right)$$

Where:

A = Amount of p‑nitrophenol produced (nmol).

V = Volume of test sample (mL).

T = Reaction time (min).

B = Total protein concentration (µg/mL), determined by BCA assay.

##### Alizarin red staining

BMSCs were seeded in 24‑well plates at a density of 2 × 10⁴ cells/well and cultured in osteogenic induction medium for 14 days prior to Alizarin Red staining.

#### In vitro angiogenesis

The angiogenic differentiation potential of EA.hy926 cells in response to hydrogel extracts was evaluated using an in vitro angiogenesis assay kit (ECM625, Millipore^®^, USA) according to the manufacturer’s protocol. Briefly, EA.hy926 cells were seeded on ECMatrix™ gel in 96‑well plates at 30,000 cells/well, with 100 µL of hydrogel extract added to each well. Tube formation was observed under an inverted microscope and photographed at 2, 4, and 8 h of culture. The tubular network structures were quantitatively analyzed using ImageJ software by counting junctions, meshes, and branches.

### Rat calvarial defect repair study

All experimental protocols were approved by the Institutional Animal Ethics Committee. When determining the sample size, reference was made to the sample size design used in previous similar studies [15, 17]. Eighteen male Sprague‑Dawley rats (12 weeks old, 380 ± 20 g body weight) [18] were used to establish calvarial defect models for evaluating the osteogenic potential of different hydrogel materials in vivo.

Surgical procedures were performed under anesthesia induced by intraperitoneal injection of 3% sodium pentobarbital. After positioning in prone position and securing on the surgical platform, the operative field was shaved and disinfected with povidone‑iodine. The mucoperiosteum was incised along the predetermined incision line with a full‑thickness sharp dissection to ensure clean wound edges. Subsequently, the mucoperiosteal flap was fully elevated in the subperiosteal plane to adequately expose the surgical site. After implantation of the gel materials, their surfaces were naturally covered by the intact periosteal layer. Given the integrity of the mucoperiosteal flap, the periosteal layer was not sutured separately. Instead, precise alignment and suturing of the mucosal incision achieved overall repositioning of the mucoperiosteal flap and tension‑free closure of the wound. Bilateral critical‑size defects (5 mm diameter) penetrating through the dura mater were created using a low‑speed trephine burr along the midline.

The defects were randomly divided into following groups (*n* = 6 defects per group): Negative control (defect only without material implantation), Control hydrogel group (G), and CS/CHWs‑OCP composite hydrogel groups (CG3, CG6, CG9, CG12). The surgical operators were aware of the group allocations (as they needed to implant different materials), while researchers performing radiographic analysis and histological evaluation were blinded to the grouping information.

Lyophilized hydrogel samples were implanted into respective defects, followed by meticulous wound closure. Postoperative care included intramuscular antibiotic administration for 3 days and standard housing conditions.

#### Micro‑CT evaluation of bone regeneration

After 8 weeks, animals were sacrificed and calvarial specimens containing the defect sites with surrounding bone tissue were harvested. Samples were rinsed with physiological saline, fixed in 4% paraformaldehyde for 48 h, and then thoroughly washed. Micro‑CT (SkyScan 1176, Bruker, USA) scanning was performed to assess new bone formation. The scanning parameters were set as follows: voltage 65 kV, current 800 µA, with a 0.5 mm aluminum filter and a rotation step of 0.3 °.

Three‑dimensional reconstruction and quantitative analysis included: Bone volume/total volume (BV/TV, %), Bone surface/total volume ratio (BS/TV, mm⁻¹), Trabecular number (Tb.N, mm⁻¹), and Trabecular separation (Tb.Sp, mm).

### Histological analysis

Specimens were decalcified in 10% EDTA solution at room temperature for 3 months. Following decalcification, samples were paraffin‑embedded, sectioned, and stained with hematoxylin‑eosin (H&E) and Masson’s trichrome. Histomorphological examination was conducted using an inverted microscope.

### Statistical analysis

Data were analyzed using SPSS 25.0 (IBM Co., Chicago, IL, USA). Normality and homogeneity of variance were verified using Shapiro‑Wilk and Levene tests, respectively. Statistical differences among groups were determined by one‑way ANOVA followed by Bonferroni between‑group comparisons. A p‑value < 0.05 was considered statistically significant.

## Results and discussion

### Characterization of the morphology and structure of hydrogels

**Fig. 1:**
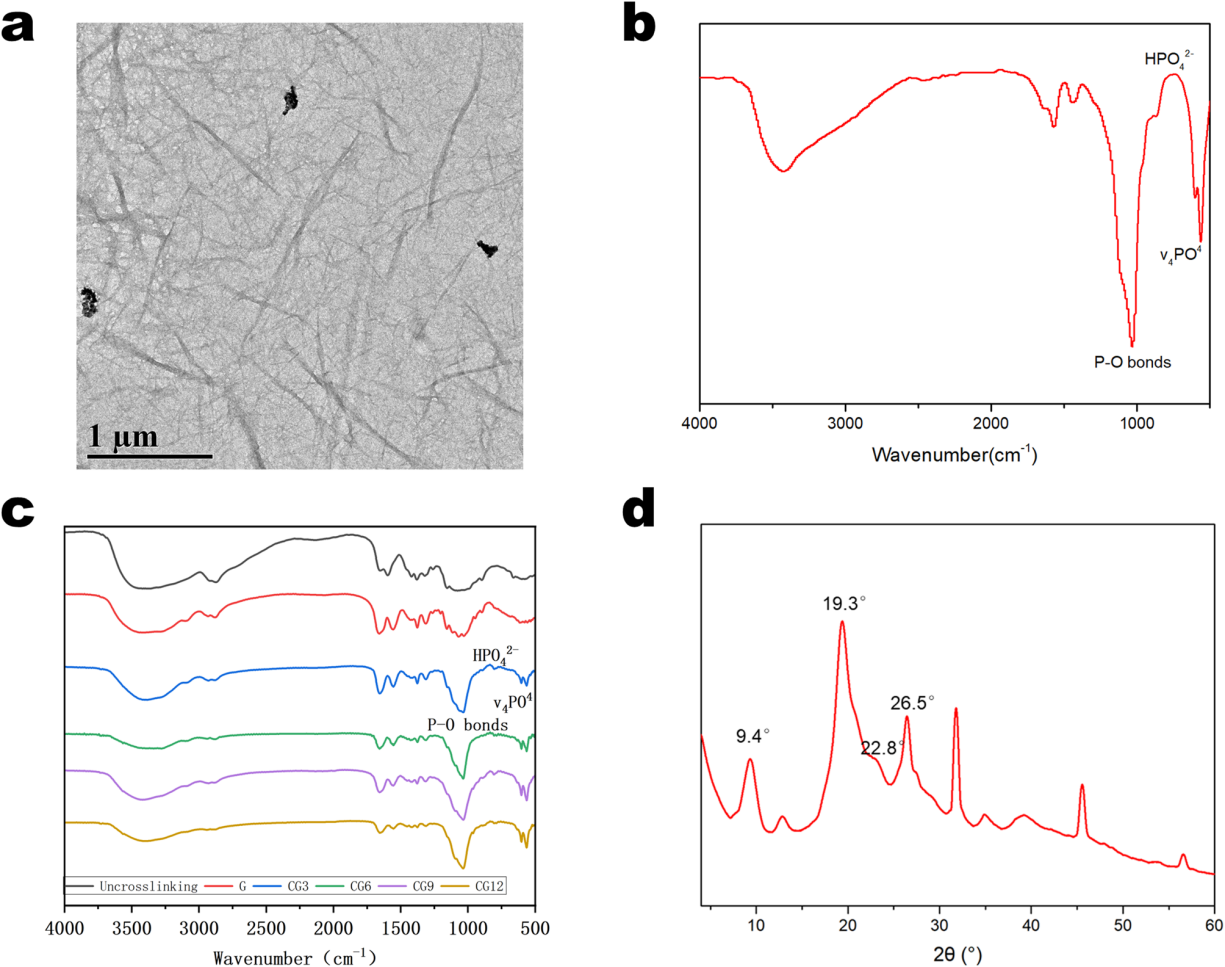
Characterization of the morphology and structure. (**a**) TEM image of CHWs. (**b**) FTIR spectra of OCP. (**c**) FTIR spectra (respectively, Uncrosslinked, G, CG3, CG6, CG9 and CG12). (**d**) XRD patterns of G hydrogel

The TEM image of chitin nanowhiskers (CHWs) (Fig. 1a) shows that the synthesized CHWs exhibit a typical rod‑like or needle‑like nanostructure, indicating their successful preparation.

The FTIR spectrum of pure OCP (Fig. 1b) exhibited a sharp P‑O band at 1023 cm⁻¹, along with HPO₄²⁻ bands at 908 cm⁻¹ and 854 cm⁻¹. Two characteristic absorption bands of crystalline calcium phosphate (ν₄PO₄) were observed between 560 and 600 cm⁻¹.

It has been widely reported in the literature that chitosan and glutaraldehyde can undergo crosslinking via a Schiff base reaction (forming C = N bonds) [19].

The FTIR spectrum of the hydrogel sample shows that after crosslinking (G), the ν(C = N) stretching vibration peak around 1640 cm⁻¹ is significantly enhanced compared to that before crosslinking (see Fig. 1c-uncrosslinking). This enhancement is attributed to the Schiff base reaction between the amino groups of chitosan and the aldehyde groups of glutaraldehyde. Meanwhile, the broad peak in the 3200–3500 cm⁻¹ region, corresponding to O–H and N–H stretching vibrations, becomes narrower and shifts after crosslinking, further confirming the reaction between amino and aldehyde groups.

Additionally, the absorption peaks in the range of 560–600 cm⁻¹, corresponding to the ν₄ bending vibration mode of phosphate groups, are crucial for assessing the phase purity of OCP as a precursor to HA and its potential transformation within the gel. OCP typically exhibits a clearly split double peak in this region (around 560 cm⁻¹ and 600 cm⁻¹), whereas HA generally presents as a broader single peak or poorly resolved shoulder peaks.

The OCP‑incorporated hydrogels displayed P‑O bands, HPO₄²⁻ bands, and two sharp absorption peaks consistent with crystalline calcium phosphate (ν₄PO₄) [20], confirming successful fabrication of CS/CHWs‑OCP composites.

The XRD pattern of G hydrogel (Fig. 1d) revealed distinct reflections at 2θ = 9.4 °, 19.3 °, 22.8 °, and 26.5 °, matching characteristic peaks of α‑chitin [21].

**Fig. 2:**
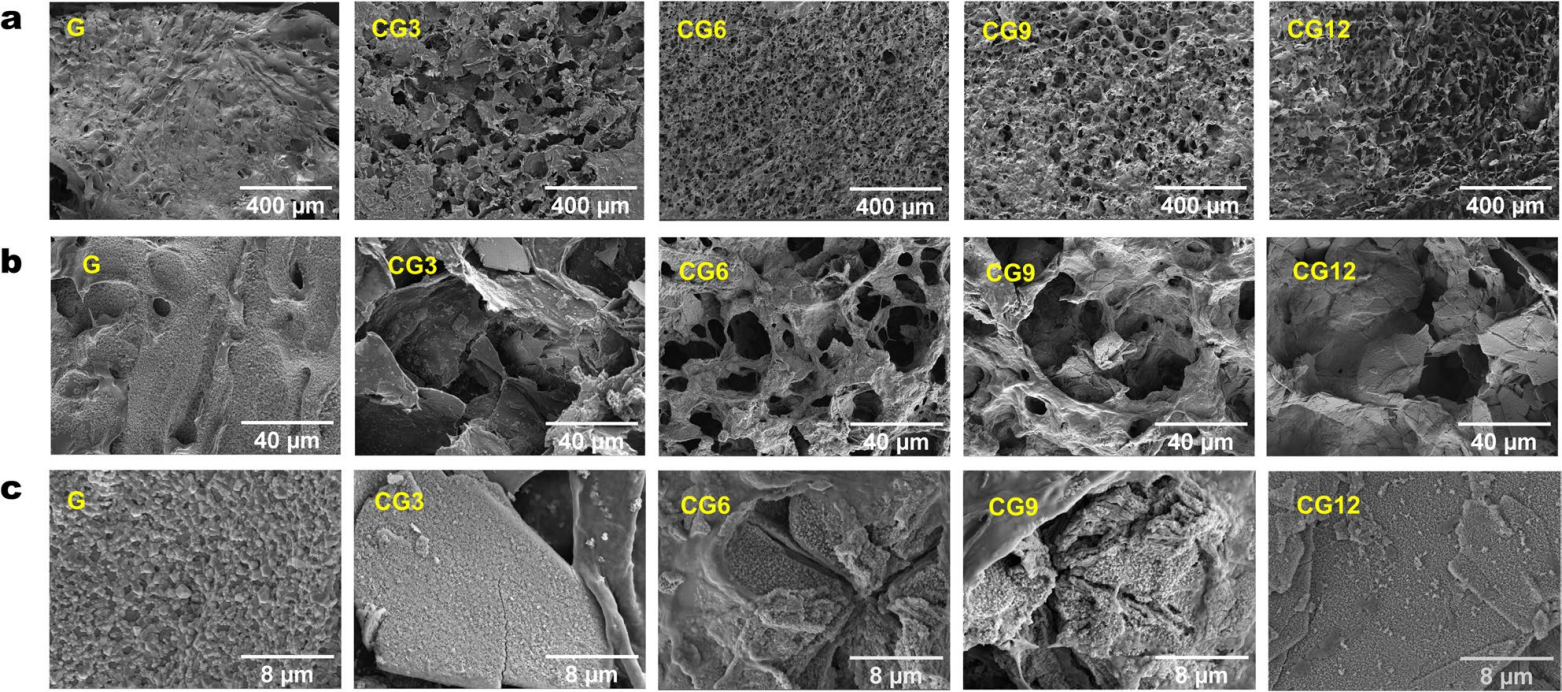
SEM images of fabricated hydrogels. (**a**) 100× (scale bar = 400 μm), (**b**) 1000× (scale bar = 40 μm), and (**c**) 5000× (scale bar = 8 μm) magnification

SEM images (Fig. 2a) demonstrated that while the G hydrogel appeared dense, all CS/CHWs‑OCP composite hydrogels (CG3‑CG12) exhibited honeycomb‑like porous structures. Higher magnification (Fig. 2b) revealed interconnected pores. Porosity values of 36.84%–56.64% were detected by ImageJ analysis, with no significant differences among OCP‑content variants. Notably, CS/CHWs‑OCP composite hydrogels (Fig. 2c, CG3‑CG12) displayed particulate surface deposits absent in the OCP‑free control (G).

It has been demonstrated that the freeze‑drying process of gels can generate pores within the scaffold through the sublimation of ice crystals, thereby forming a porous structure conducive to cell growth and tissue regeneration [22, 23]. SEM results of the CS/CHWs‑OCP composite hydrogels indicate that the prepared hydrogels in this study possess interconnected porous structures. Such microporous architectures in the scaffolds facilitate the guided flow of proteins and may provide essential conditions for the transport of nutrients and metabolic waste [24, 25].

### Mechanical testing

**Fig. 3:**
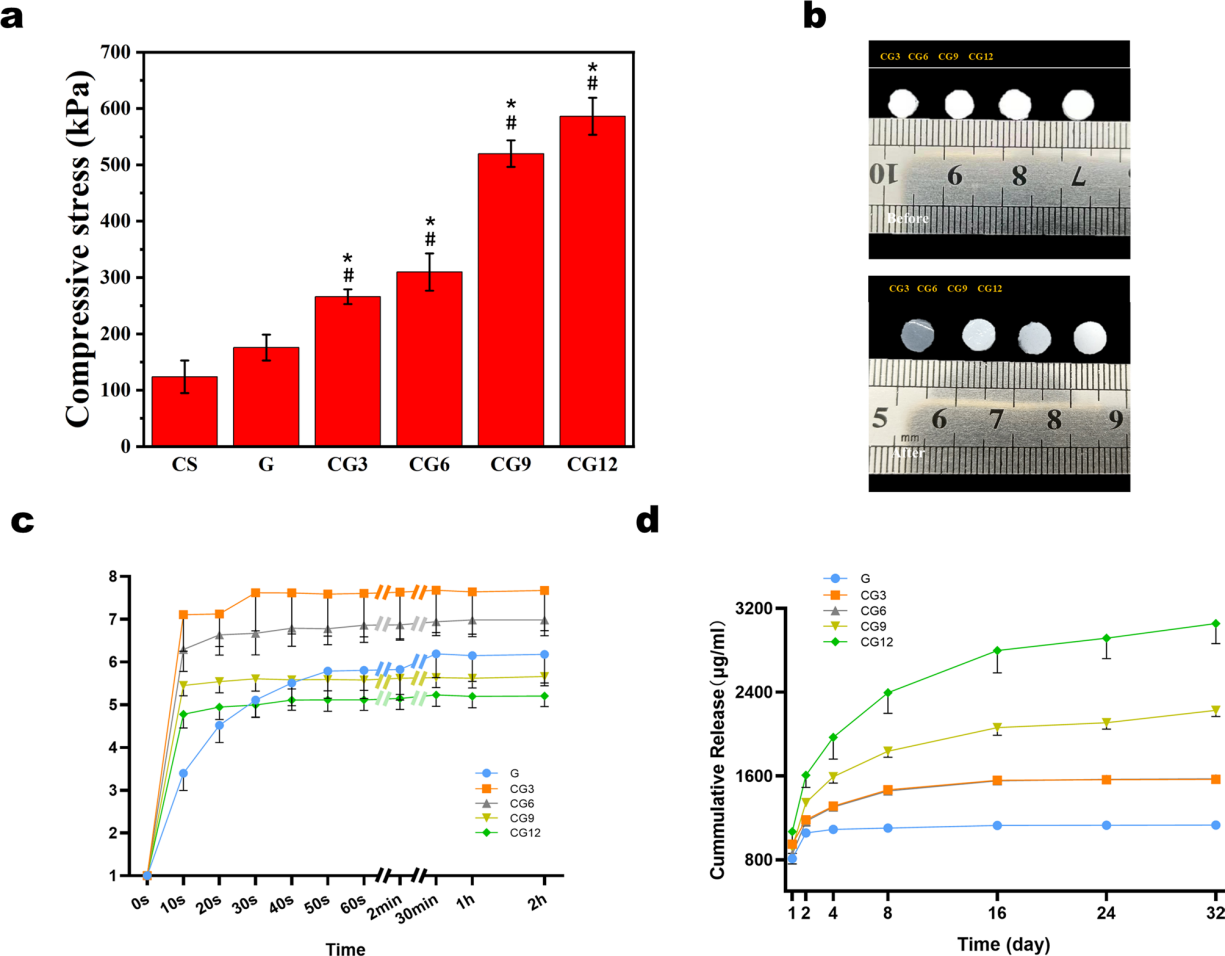
Physico‑chemical characterization of prepared hydrogels. (**a**) Compressive stress of hydrogels. (**b**) Hydrogels before and after swelling. (**c**) Swelling curves of different hydrogel groups. (**d**) In vitro BSA release profiles from hydrogels in PBS. (#, vs. Group CS (without CHWs): # *P* < 0.05; *, vs. Group G (with CHWs): * *P* < 0.05)

We evaluated the mechanical properties of the hydrogels through compression testing. The results indicate that while the addition of CHWs alone provides limited improvement in compressive strength, their combination with OCP demonstrates a significant synergistic enhancement effect (Fig. 3a). We hypothesize that this phenomenon arises from the dual functionality of CHWs within the system: firstly, as nano‑reinforcing fibers that preliminarily strengthen the CS network; and secondly, more importantly, their unique surface chemistry and nanofiber morphology mimic the templating role of natural collagen fibers. CHWs effectively adsorb calcium ions, guiding the orderly nucleation and growth of OCP on their surfaces, thereby regulating the crystallization process of the bioactive inorganic phase. This controlled mineralization process enables OCP crystals to form strong interfacial bonding and a highly integrated microstructure with the organic matrix (CS/CHWs), rather than randomly distributed secondary phase aggregates. Consequently, the resulting ternary composite hydrogel not only exhibits significantly enhanced compressive strength but also likely possesses excellent bioactivity and biomimetic mineralization capabilities. This work suggests that CHWs in such biomimetic mineralization systems are not merely mechanical fillers but are key guiding components that enable synergistic organic–inorganic assembly and impart bioactivity to the material.

### Swelling properties

As shown in Fig. 3b, all CS/CHWs‑OCP hydrogels maintained dimensional stability with < 20% diameter change post‑swelling.

Swelling capacity is critical for hydrogel scaffolds. Moderate swelling maintains mechanical/structural stability [26] while expanding internal pores for cell infiltration [27].

Our hydrogels achieved 5–8‑fold weight increase in PBS (Fig. 3c), indicating excellent hydrophilicity and potential as growth factor/drug carriers. All groups reached swelling equilibrium rapidly.

There is a non‑linear relationship between the amount of octacalcium phosphate added and the swelling performance of the hydrogel. The reason for this may be that octacalcium phosphate plays a dual role in the gel. When the addition amount is low, OCP particles primarily act as dispersed physical crosslinking points, which, while increasing the crosslinking density of the network, also introduce structural heterogeneity, thereby inhibiting the uniform swelling of the gel and leading to a decrease in the swelling ratio. In contrast, when the addition amount is high, OCP particles form interconnected hydrophilic inorganic networks that, together with the chitosan organic network, create a synergistically reinforced composite structure. On one hand, this significantly enhances the overall hydrophilicity and water absorption capacity of the material. On the other hand, the rigid inorganic skeleton provides stable support for the gel, allowing it to maintain structural integrity even under high swelling conditions, thereby demonstrating a swelling capacity that surpasses that of purely organic gels.

However, excessive swelling may cause tissue compression and impair cell adhesion. Our hydrogels showed minimal volumetric changes (Fig. 3c), demonstrating superior dimensional stability that avoids mechanical stress on surrounding tissues.

### Protein release test

BSA is commonly used as a release model for macromolecular drugs/growth factors to preliminarily evaluate the drug‑loading and release capabilities of composite hydrogels, owing to its stable physicochemical properties, well‑defined molecular weight (~ 66 kDa), and ease of detection. In this study, a concentration of 5 mg/mL BSA was selected to ensure accurate detection via UV‑Vis spectroscopy.

The protein release test results indicate that higher OCP content leads to an accelerated release rate. This may be attributed to the fact that a higher content of OCP particles likely creates more interconnected channels or micropores within the three‑dimensional network of the hydrogel, providing more accessible pathways for BSA diffusion and thus manifesting as “accelerated release.” This observation differs from the traditional notion where inert fillers slow down release, highlighting the active dissolution and pore‑forming role of OCP in this system. We regard this as a distinctive feature of the release behavior in this composite material and plan to further validate it in future studies.

The control hydrogel (G) reached a release plateau within 2 days, whereas CS/CHWs‑OCP composites (CG3‑CG12) sustained protein release over 32 days (Fig. 3d).

Sustained bioactive molecule release is crucial for continuous osteogenesis. Literature suggests optimal release durations between 2 and 4 weeks for bone regeneration factors [28]. Therefore, this study established a 32‑day release cycle to investigate whether the release duration of BSA protein aligns with the optimal release period.

The composite gels prepared in this study exhibit protein release kinetics similar to those reported in the literature, suggesting their potential advantage as bone regeneration scaffolds with sustained‑release functionality.

However, given the significant difference in molecular weight between BSA and most bone regeneration‑related growth factors (such as BMP‑2, ~ 26 kDa), future studies are required to further examine the release profiles and biological performance of bone regeneration‑related growth factors when loaded in the system.

### In vitro biological performance

#### Material biocompatibility

**Fig. 4:**
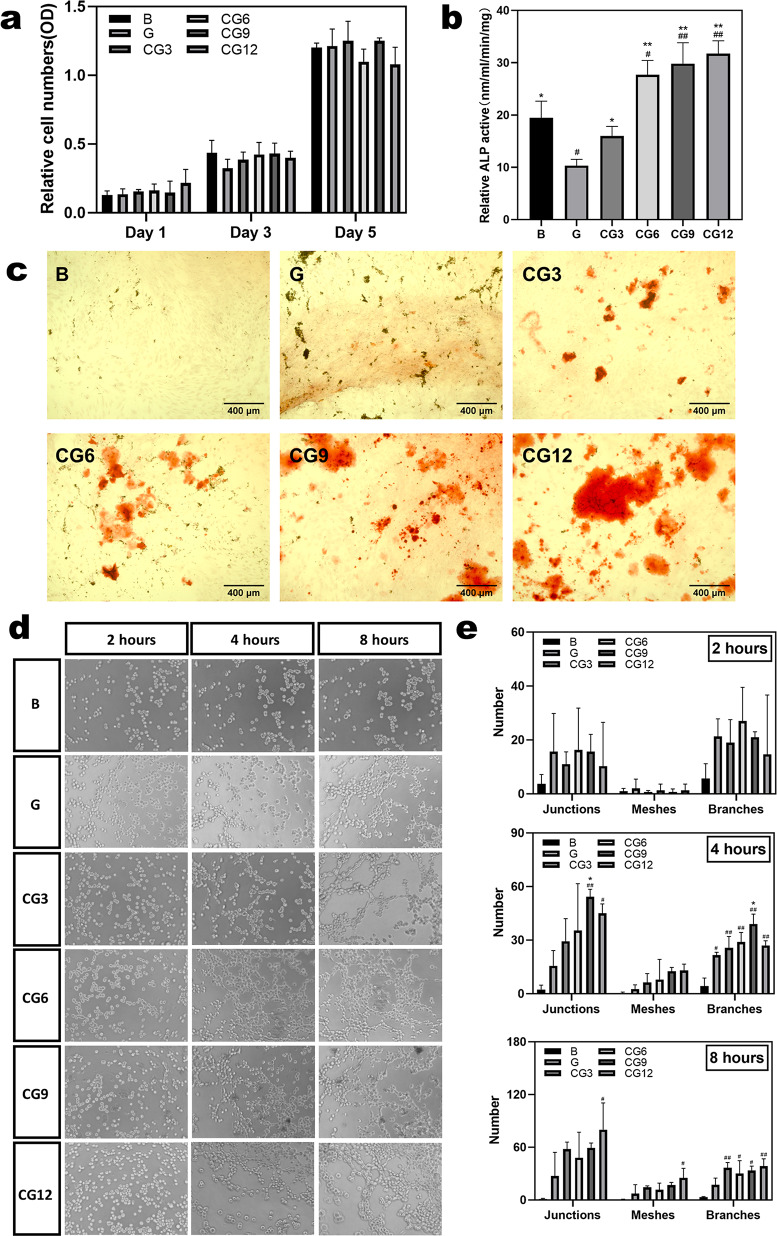
Biocompatibility, osteogenic and angiogenic differentiation. (**a**) CCK‑8 assay of BMSCs cultured with hydrogels for 1, 3, and 5 days, respectively. (**b**) ALP activity of BMSCs cultured with different hydrogels for 7 days. (**c**) Alizarin red staining after 14 days of culture. Scale bar = 400 μm. (**d**) Microscopic images of EA.hy926 cells cultured in hydrogel extracts for 2, 4, and 8 h. (**e**) Quantitative analysis of tube formation by EA.hy926 cells on ECMatrix gel after 2, 4, and 8 h, including junctions, meshes, and branches. (#, ## vs. blank control (Group B): # *P* < 0.05, ## *P* < 0.01; *, ** vs. control group (Group G): * *P* < 0.05, ** *P* < 0.01)

Figure 4a presents the proliferation profile of BMSCs co‑cultured with hydrogels for 1, 3, and 5 days. The increasing OD values over time indicate continuous cell proliferation. No statistically significant differences in OD values were observed among different groups at each time point, demonstrating excellent biocompatibility of the fabricated CS/CHWs‑OCP composite hydrogels.

#### ALP activity

As tissue scaffolds cannot directly function as recipient tissues, their osteogenic process primarily involves fusion with host tissue followed by gradual replacement through neo‑tissue formation [29]. This complex process encompasses multiple critical stages, including recruitment and adhesion of osteogenic cells, osteogenic differentiation, bone matrix formation, and mineralization [30]. Therefore, ideal bone scaffold materials should not only exhibit good cytocompatibility but also actively promote osteogenic differentiation of relevant cells.

ALP, an enzyme expressed during osteogenic differentiation, serves as an important marker for early‑to‑mid stage osteogenic differentiation capacity of BMSCs. Our ALP activity results (Fig. 4b) demonstrate that CS/CHWs‑OCP composite hydrogels possess osteoinductive potential that correlates with OCP content, consistent with findings by Saito et al. [31].

Notably, the blank control group (B) showed higher ALP expression than the hydrogel control (G), which may be attributed to the inherent osteogenic potential of BMSCs and potential morphological/porosity constraints of the pure hydrogel. The significantly enhanced ALP activity in CS/CHWs‑OCP groups compared to the G group likely stems from direct effects of incorporated OCP crystals. Previous studies have established that OCP crystals can upregulate ALP activity and osteopontin expression while supporting new bone matrix formation by osteoblasts [32, 33].

#### Alizarin red staining

Calcium deposition, reflecting late‑stage osteogenic capacity, serves as a hallmark of osteoblast maturation in vitro. Figure 4c displays Alizarin Red staining results of BMSCs cultured on control (G) and composite hydrogels (CG3‑CG12) for 14 days. Quantitative analysis revealed significantly more calcium nodules in composite hydrogel groups compared to the G group, with nodule formation positively correlated to OCP content [34]. These findings further confirm the OCP dose‑dependent osteogenic promotion capacity of our composite hydrogels.

#### In vitro angiogenesis

The angiogenic potential of EA.hy926 cells was evaluated using an in vitro angiogenesis kit with extracts from different composite hydrogels.

We employed extraction rather than direct culture to preliminarily evaluate the overall biocompatibility of the material extract and its potential to induce paracrine effects. This approach excludes the direct contact‑guided influence of the material’s surface physical topography, focusing instead on the impact of degradation products (such as calcium and phosphate ions) from the OCP component in the composite material. This setup more closely simulates the early changes in the local microenvironment following material implantation.

During early and intermediate stages of vascularization, EA.hy926 cells formed tubular structures on ECMatrix™ gel. Results demonstrated that CG6, CG9, and CG12 groups initiated tube formation as early as 4 h, whereas G and CG3 groups required 8 h, with CG3 exhibiting more extensive networks than G. No significant tubular structures were observed in blank controls at any time point (2/4/8 h, Fig. 4d). These findings suggest that CS/CHWs‑OCP composites promote EA.hy926 vascularization, with OCP incorporation further enhancing this effect in a dose‑dependent manner. Previous studies indicate that OCP‑containing culture medium can directly stimulate angiogenesis in vascular endothelial cells [15]. The high chemical solubility of OCP facilitates calcium ion release, and calcium signaling has been reported to critically regulate HUVEC migration, proliferation, and in vivo angiogenesis [35]. Research by Wang et al. [36] demonstrated that Ca²⁺ released from the degradation of calcium phosphate (CaP) bioceramics activates the Calcineurin (CaN) signaling pathway via Store‑Operated Ca²⁺ Entry (SOCE) channels. This promotes nuclear translocation of the transcription factor NFATc1, ultimately upregulating the expression and secretion of vascular endothelial growth factor (VEGF), thereby inducing angiogenesis. Furthermore, recent studies have indicated that OCP and its composites (e.g., with gelatin) can effectively promote angiogenesis during new bone formation. The specific mechanisms involve stimulating the formation of certain vascular subtypes (such as H‑type vessels) and highlight the dual “vascular‑osteogenic” capability of OCP [15, 37]. These may partially explain the pro‑angiogenic properties of CS/CHWs‑OCP composites.

However, these findings remain indirect evidence. In the future, further validation through qPCR or ELISA is required to assess the gene or protein expression of key pro‑angiogenic factors (such as VEGF and bFGF) in endothelial cells treated with the hydrogel extract, in order to elucidate the angiogenic mechanism of this gel material.

### In vivo bone repair performance

The 5 mm diameter rat cranial defect, which cannot be healed spontaneously, is considered a classic critical‑sized bone defect model and has been widely used in studies on various bone regeneration scaffold materials [38].

#### Micro‑CT evaluation

Quantitative analysis was performed using CTAn software (Version 1.16.9.0). For each sample, a cylindrical volume of interest with a diameter of 5.0 mm was defined within the defect region for analysis. By examining the grayscale histogram of representative samples, a fixed global threshold (grayscale value: 92, corresponding to the 8‑bit image range of 0–255) was applied to separate mineralized bone tissue from the background. The binarized images were subsequently subjected to 3D despeckling (removing isolated islands smaller than 10 pixels) and a single closing filter with a 3 × 3 × 3 kernel. The following parameters were calculated: bone volume/total volume ratio (BV/TV), bone surface area/total volume ratio (BS/TV), trabecular number (Tb.N), and trabecular separation (Tb.Sp).

**Fig. 5:**
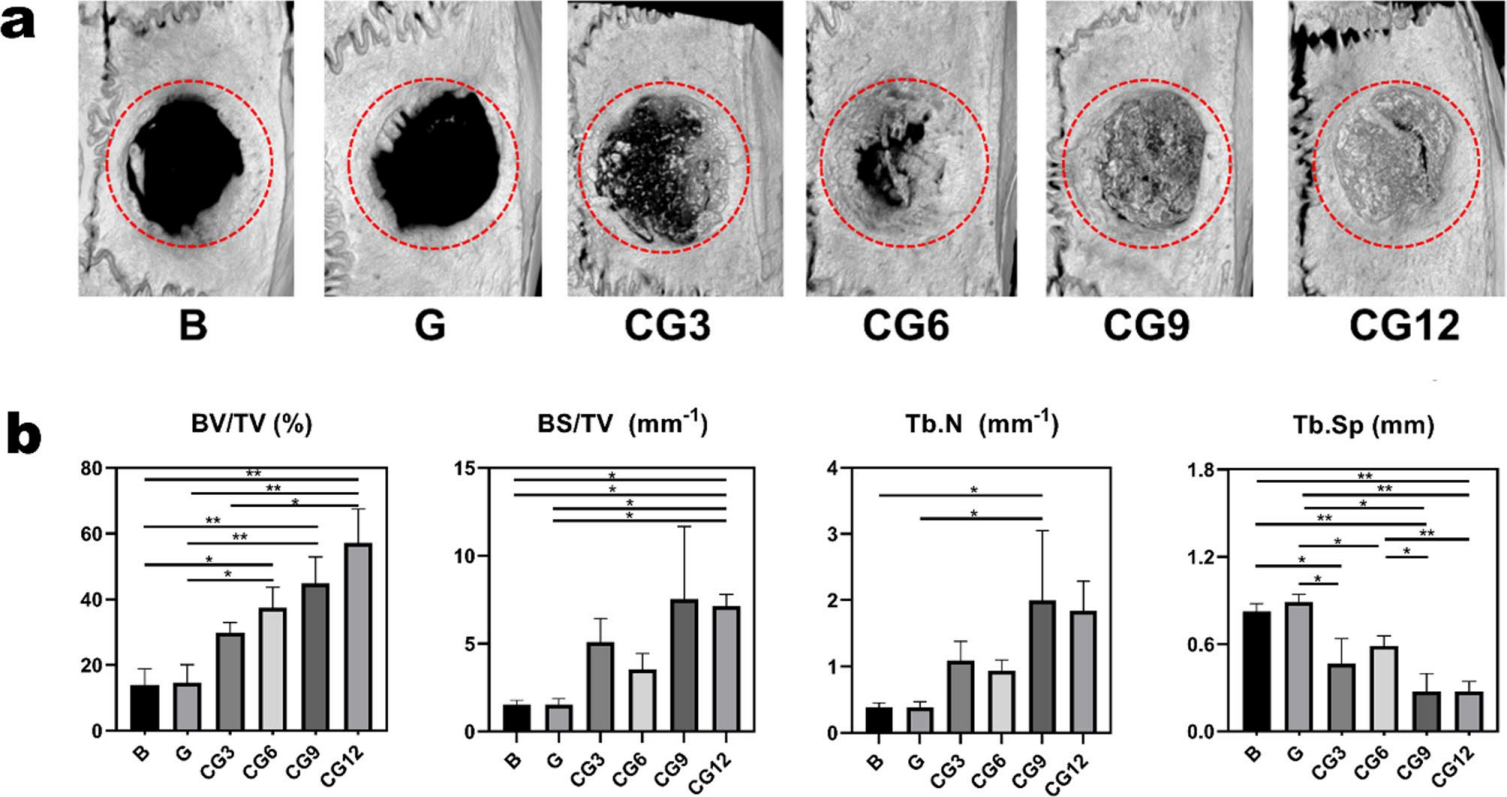
Micro-CT analysis (**a**) Three-dimensional micro-CT reconstruction images of rat cranial defects 8 weeks after hydrogel implantation. (**b**) Quantitative analysis of new bone formation within the defect areas, including: bone volume/total volume ratio (BV/TV), bone surface area/ total volume ratio (BS/TV), the number of beams (Tb.N) and the separation of trabecular bones (Tb.Sp) (^*^P < 0.05, ^**^P < 0.01; data presented as mean ± SD)

Micro‑CT analysis of cranial defect repair revealed that the 5‑mm critical‑sized defects in rat calvaria failed to undergo spontaneous healing, with only minimal new bone formation observed at the defect margins, confirming the successful establishment of the critical‑sized defect models. In contrast, the CS/CHWs‑OCP composites (CG3, CG6, CG9, CG12) exhibited varying degrees of high‑density signals in the central region of the defects, indicating new bone formation. These findings demonstrate that CS/CHWs‑OCP composites significantly enhanced osteogenesis in the calvarial defects. Furthermore, the volume of newly formed bone increased proportionally with the OCP content (Fig. 5a).

The evaluation of new bone formation in 5 mm diameter cranial defects is illustrated in Fig. 5b. BV/TV: No significant difference was observed between the blank group (B) and the control gel group (G). However, the BV/TV values in the cranial defects implanted with composite gels (CG6, CG9, CG12) were significantly higher than those in groups B and G. Although no statistical difference was detected in the CG3 group, an increasing trend was still evident compared to the blank and G groups. Among the CS/CHWs‑OCP composites, the BV/TV value increased with the proportion of OCP. Additionally, BS/TV showed an upward trend, indirectly indicating a gradual increase in osteogenic volume within the region. Similarly, Tb.N exhibited an increasing tendency in the CS/CHWs‑OCP composites. Tb.Sp demonstrated a decreasing trend, with all CS/CHWs‑OCP composites being significantly lower than the control gel group (G). The reduction in the average width between trabeculae further confirmed enhanced new bone formation. Among the CS/CHWs‑OCP composites with varying OCP content, Tb.Sp in CG9 and CG12 was significantly lower than in CG6, suggesting greater new bone formation in the CG9 and CG12 groups. These results indicate that, with the support of the gel scaffolds, osteoblasts can grow, proliferate, differentiate, and mineralize on the scaffolds’ surface or within their interior, enabling concurrent new bone formation at the defect center rather than being confined solely to the peripheral regions.

However, it should be noted that the high‑density signals observed in Micro‑CT images correspond to “newly formed bone and/or undegraded scaffold material.” The definitive identification of newly formed bone primarily relies on histological findings.

#### Histological evaluation

**Fig. 6:**
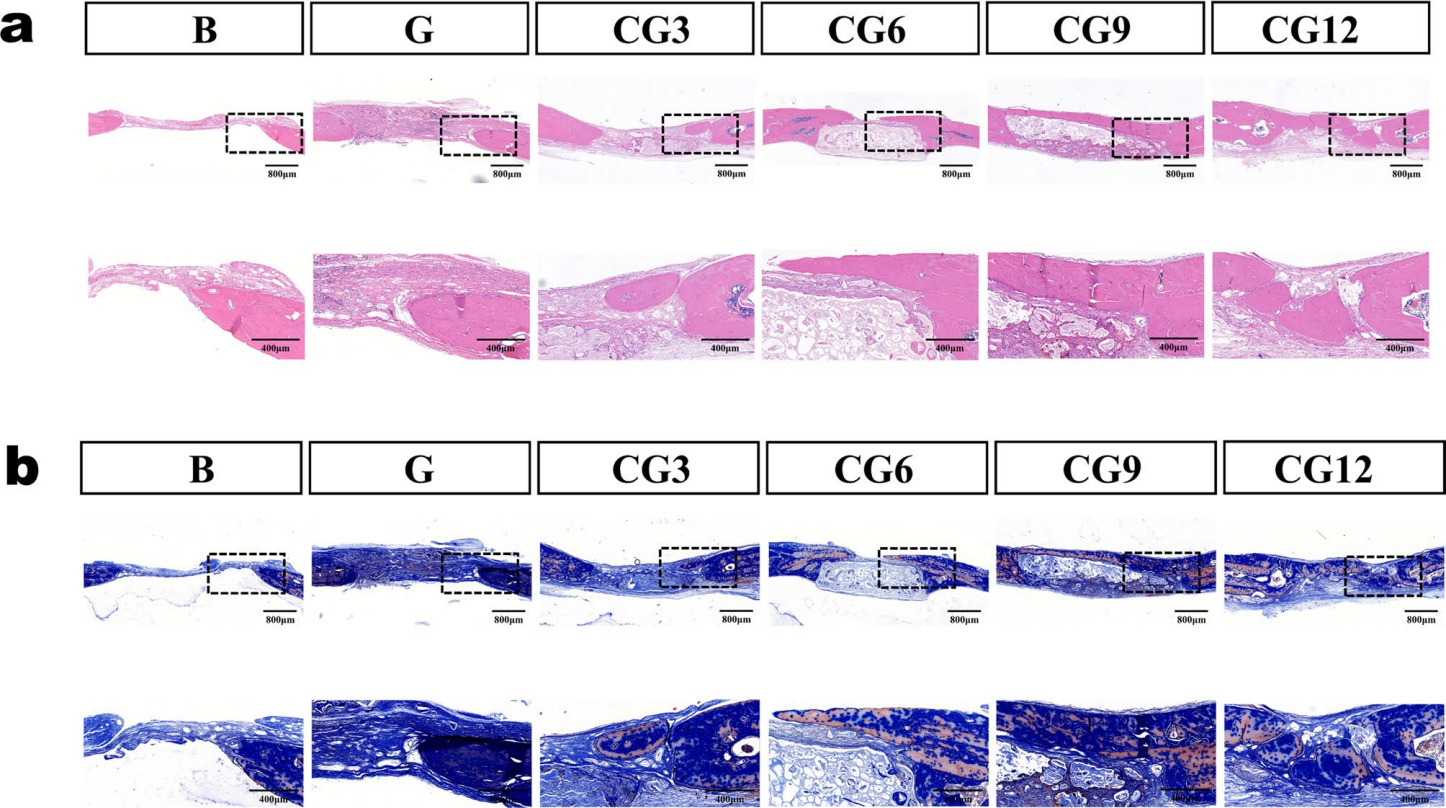
Histological assessment of cranial bone regeneration. (**a**) H&E staining of in vivo osteogenesis of different hydrogels at 8 weeks post‑implantation (sagittal plane). (**b**) Masson’s trichrome staining of bone formation in different scaffolds at 8 weeks (sagittal plane)

The results of H&E staining and Masson’s trichrome staining (Fig. 6) show that the central area of the bone defect in the blank group is primarily composed of loose connective tissue, while the central area in the G group is mainly occupied by residual gel material. In contrast, the defect areas treated with composite materials show the presence of newly formed bone tissue, albeit with uneven thickness. The CS/CHWs‑OCP composite group exhibits more new bone formation, with the CG12 gel group displaying the most abundant osteogenesis. Additionally, the blank group experienced collapse of the defect area due to a lack of effective support, whereas the CS/CHWs‑OCP composite groups maintained good contour integrity. This indicates that the material possesses favorable mechanical support and osteoconductive properties, consistent with the micro‑CT observations.

It should be noted that the scaffold structures in different CG groups appear unevenly distributed, which is primarily attributed to differences in the degradation of the materials in vivo. Furthermore, the new ossified tissues formed at the defect site are mainly distributed above or below the scaffold and do not infiltrate the scaffolds.

The reasons for this may include: at the relatively early time point of 8 weeks, new bone formation tends to initiate from the well‑vascularized host bone edges and periosteal side via “creeping substitution”; meanwhile, vascularization and cellular infiltration in the central region of the scaffold require more time. Additionally, the space‑occupying effect of OCP particles within the scaffold may have temporarily hindered complete cellular migration. These observations suggest that further optimization of the internal pore connectivity and degradation rate of the scaffold will be an important direction for future research.

Although this study has preliminarily demonstrated the excellent short‑term bone regeneration efficacy of the CS/CHWs/OCP composite scaffolds, its long‑term stability (≥ 6 months) in large animal models (e.g., mandibular defects in beagle dogs) still requires further validation.

### Strengths of the study

This study presents several key strengths in addressing the critical challenge of balancing mechanical integrity and bioactivity in bone repair scaffolds:

#### A novel synergistic strategy 

We propose and validate an innovative “mechanical reinforcement–biomineralization” synergistic strategy. This approach moves beyond single‑property enhancement by concurrently strengthening the hydrogel network and introducing bioactive mineralization.

#### A rational and functional material design 

A novel chitosan‑based composite hydrogel was constructed, co‑modified with chitin nanowhiskers (CHWs) and octacalcium phosphate (OCP) with gradient loadings (3–12 wt%). The design is rational: CHWs provide nanoscale reinforcement and templating for mineralization, while OCP, chosen over the more inert hydroxyapatite (HA), acts as a highly bioactive and biodegradable calcium‑phosphate source to enhance the osteogenic microenvironment.

#### Comprehensive bioactivity validation 

The dual functionality of the CS/CHWs‑OCP hydrogels was systematically demonstrated. They exhibit robust in vitro bioactivity by promoting both osteogenesis (in BMSCs) and angiogenesis (in EA.hy926 cells). More importantly, their excellent in vivo bone defect repair efficacy was preliminarily confirmed in an animal model, bridging the gap between material design and biological performance.

#### High translational potential 

By integrating superior mechanical support, controllable biodegradation, and proven osteogenic‑angiogenic coupling, this work provides a promising and translatable scaffold solution for advanced bone tissue engineering.

### Limitations and future perspectives

#### Characterization of nanofiller dispersion state and stability

Current research primarily relies on macroscopic mechanical and biological performance evaluations. While the zeta potential of CHWs and their precise dispersion state within the matrix are critical for their reinforcing effect, interfacial interactions with OCP, and long‑term stability in the hydrogel, these parameters were not systematically investigated in this study. Future work should incorporate such characterizations, employing higher‑resolution imaging techniques (e.g., high‑magnification SEM or TEM) combined with spectroscopic analysis to directly visualize the nanoscale dispersion of CHWs and their interactions with OCP crystals, thereby establishing a clearer structure–performance relationship.

#### Protein release characteristics and bioactivity validation

Although the sustained‑release capability of the hydrogel was demonstrated using bovine serum albumin (BSA) as a model protein, its molecular weight differs significantly from key osteogenic or angiogenic growth factors (e.g., BMP‑2, VEGF). Future studies should further evaluate the loading efficiency, release kinetics, and—most importantly—the retention of bioactivity of relevant growth factors loaded in this hydrogel, to more accurately predict its therapeutic efficacy.

#### Degradation performance of the material

Biodegradability is a key characteristic of bone repair materials, with an ideal degradation rate matching the rate of new bone formation to provide dynamic space for cell migration and tissue remodeling. While the in vitro mineralization capacity, mechanical support, and bioactivity of the material were systematically evaluated in this study, the long‑term in vivo degradation behavior of the CS/CHWs‑OCP hydrogel was not systematically examined. In future work, longer‑term animal experiments combined with histological methods will be designed to quantitatively investigate the degradation kinetics of this material at the defect site and its spatiotemporal coupling with the new bone formation process, thereby comprehensively evaluating its long‑term performance as a biodegradable bone repair scaffold.

#### Quantitative histomorphometric analysis and sample size

Although the in vivo bone regeneration results are encouraging, the limited sample size restricts this study to a preliminary exploratory investigation. To enhance statistical power and reliability, future studies should employ a larger animal sample size and perform more rigorous quantitative histomorphometric analysis of the newly formed bone, including parameters such as bone mineral density (BMD), trabecular thickness, and connectivity.

#### Investigation of osteogenic–angiogenic synergistic mechanisms

This study provides functional evidence of enhanced bone and vascular formation, but the underlying molecular mechanisms remain insufficiently explored. Future work should focus on mechanism‑oriented studies at the gene and protein expression levels to uncover how this composite hydrogel coordinates osteogenic–angiogenic coupling. This fundamental understanding will provide a more solid theoretical basis for the clinical translation and rational optimization of such biomaterials.

## Conclusion

We successfully fabricated porous CS/CHWs‑OCP composite hydrogels with sustained‑release capacity. These materials exhibited excellent BMSC compatibility, dual osteogenic‑angiogenic induction capability, and enhanced bone regeneration. These findings position CS/CHWs‑OCP composites, particularly CG12, as promising potential bone regeneration scaffold materials. 

## Data Availability

Data availability Data will be made available on request.
